# Supplementary material for: First Evidence for a Role of Siglec-8 in Breast Cancer
Source: Int J Mol Sci. 2021 Feb 18;22(4):2000. doi: 10.3390/ijms22042000 (PMC7922794; doi:10.3390/ijms22042000)
Supplement: Supplementary file 1 [file ijms-22-02000-s001.pdf]

Supplementary Information

Tables:

Table SI 1: Correlation analysis (Spearman-Rho) of Siglec-8 expression (measured by IRS) with clinical and pathological parameters and the known prognostic factors Gal-7 and TA-MUC1. Significant correlations are displayed in bold. **. Correlation is significant at the 0.01 level (2-tailed). *. Correlation is significant at the 0.05 level (2-tailed). CC = correlation coefficient. IRS = immune reactivity score. NST = no special type. HER2 = human epidermal growth factor receptor. ER = estrogen receptor. PR = progesterone receptor. N = number of patients. Gal = galectin. TA-MUC1 = tumor-associated mucin-1.

		Siglec-8 expression (IRS)
**NST vs non-NST**	CC	**0.180****
	Significance (2-tailed)	0.009
	N	213
Biological subtype	CC	0.003
	Significance (2-tailed)	0.968
	N	226
Grading	CC	0.152
	Significance (2-tailed)	0.059
	N	156
HER2 amplification	CC	0.109
	Significance (2-tailed)	0.103
	N	224
**ER-status**	CC	**0.147***
	Significance (2-tailed)	0.027
	N	226
PR-status	CC	0.110
	Significance (2-tailed)	0.098
	N	226
Tumor size (pT)	CC	0.067
	Significance (2-tailed)	0.316
	N	225
Lymph node status (pN)	CC	0.028
	Significance (2-tailed)	0.679
	N	217
Age	CC	-0.078
	Significance (2-tailed)	0.243
	N	226
	CC	**0.298****

**Gal-7 IRS (cytoplasm)**	Significance (2-tailed)	<0.001
	N	212
**TA-MUC1 IRS (cytoplasm)**	CC	**0.140***
	Significance (2-tailed)	0.039
	N	218
TA-MUC1 IRS (membranous)	CC	0.017
	Significance (2-tailed)	0.803

Figures:

<u>Figure SI1:</u>

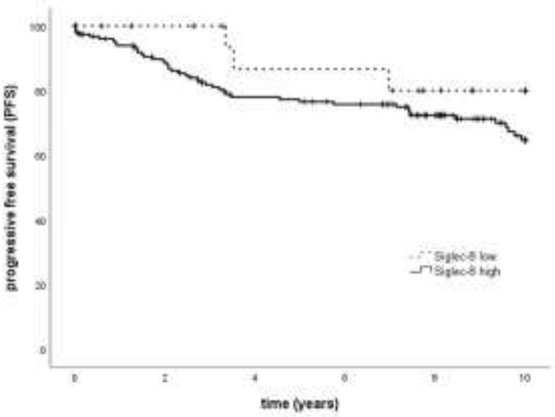

(**a**)

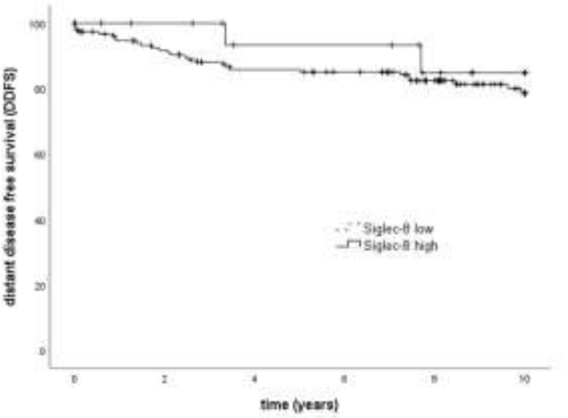

(**b**)

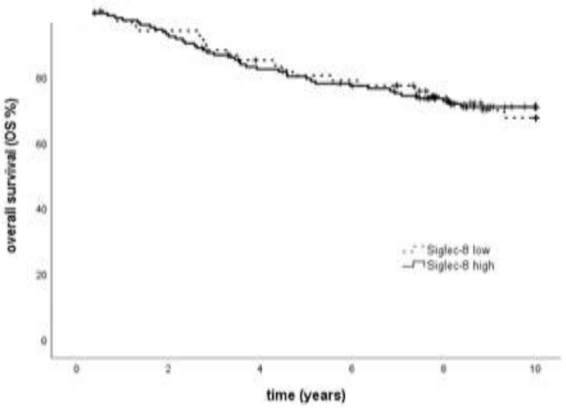

(**c**)

Figure SI 1. Association of Siglec-8 expression to the clinical outcome.
Kaplan-Meier analysis of PFS ($p = 0.971$) (a) and DDFS ($p = 0.941$) (b) and OS ($p = 0.850$) (c) in Siglec-8 high and low expressing tumors is shown. Tumors with high Siglec-8 expression show a tendentially but not significantly impaired PFS and DDFS.

Figure SI 2:

pN0 > 0

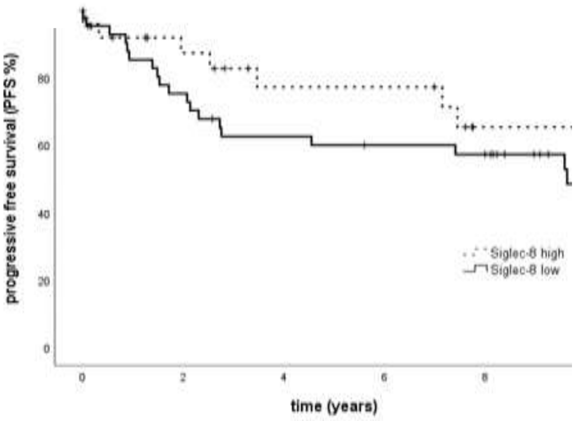

(**a**)

pN0 > 0

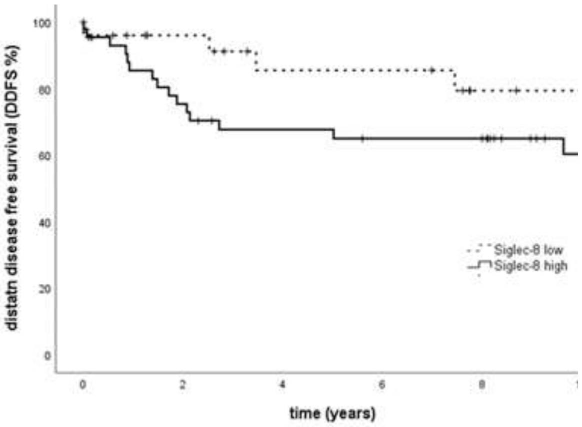

(**b**)

HER2 positive

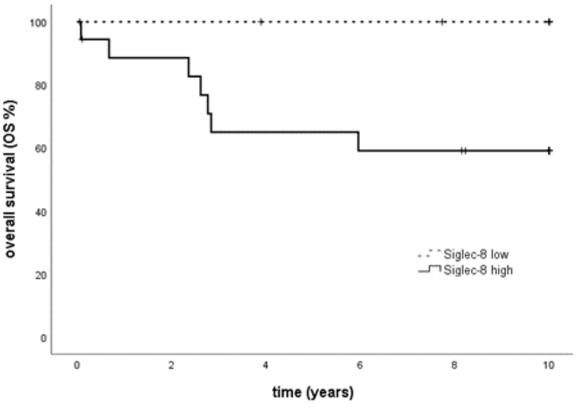

(**c**)

ER negative

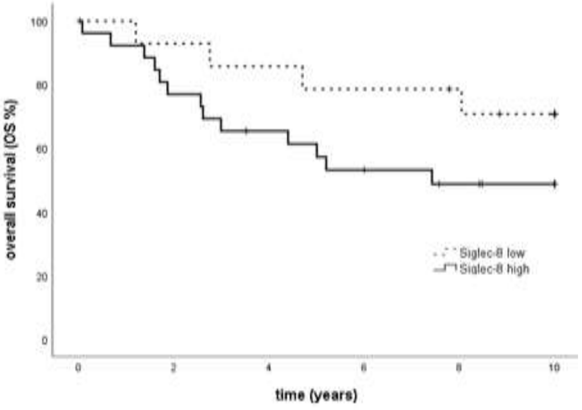

(**d**)

pN = 0

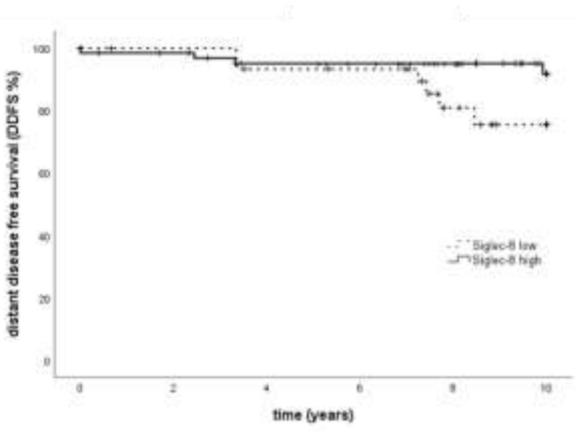

(e)

Figure SI 2. Associations of Siglec-8 expression to the clinical outcome in different subgroups with borderline significance.

Kaplan-Meier analysis of PFS (a, e), DDFS (b) and OS (c, d) in Siglec-8 high and low-expressing tumors with pN > 0 (a, b), pN = 0 (e), HER2+ (c) and ER- (d) is shown. In node-positive disease, high Siglec-8 expression is associated with a tendentially impaired PFS (p = 0.237, a) and DDFS (p = 0.117 ,b). In HER2-positive (p = 0.118, c) and ER-negative (p =0.153, d) disease, high Siglec-8 expression is tendentially associated with an impaired OS. In contrast, in node-negative BC, high Siglec-8 expression is tendentially associated with an improved DDFS (p = 0.061, e). PFS = progression free survival, DDFS = distant disease free survival, OS = overall survival, pN = lymph node status, HER2 = human epidermal growth factor receptor-2, ER = estrogen receptor.

Figure SI 3:

Siglec-8 low

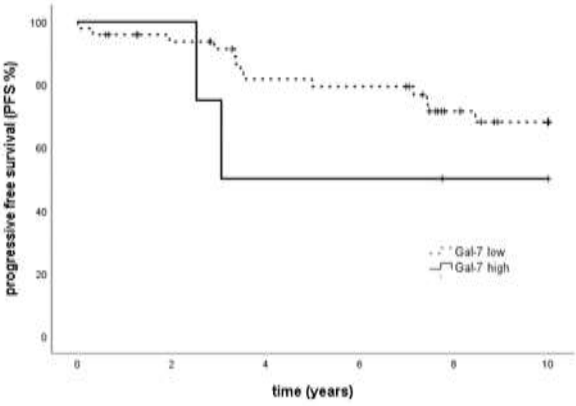

(**a**)

Siglec-8 low

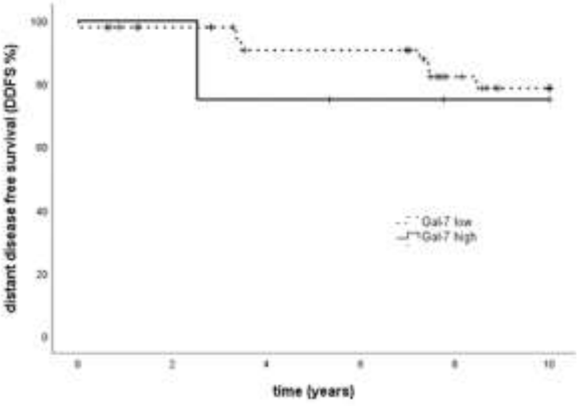

(**b**)

Siglec-8 low

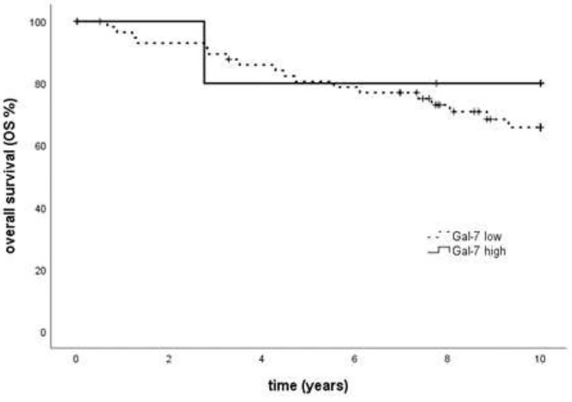

(**c**)

Siglec-8 high

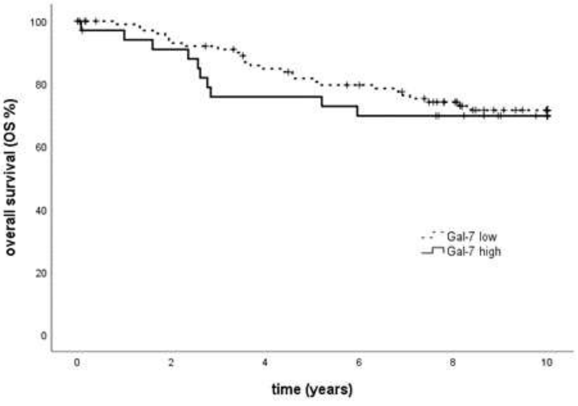

(**d**)

Figure SI 3. Not significant associations of Gal-7 expression to the clinical outcome in Siglec-8 low or Siglec-8 high subgroups.
Kaplan-Meier analysis of PFS (a) (p= 0.276), DDFS (b) (p = 0.589) and OS (c) (p = 0.632) and (d) (p = 0.653) in Gal-7 high and low-expressing tumors in Siglec-8 low-expressing (a, b, c), and Siglec-8 high expressing tumors (d).

Figure SI 4:

Siglec-8 low

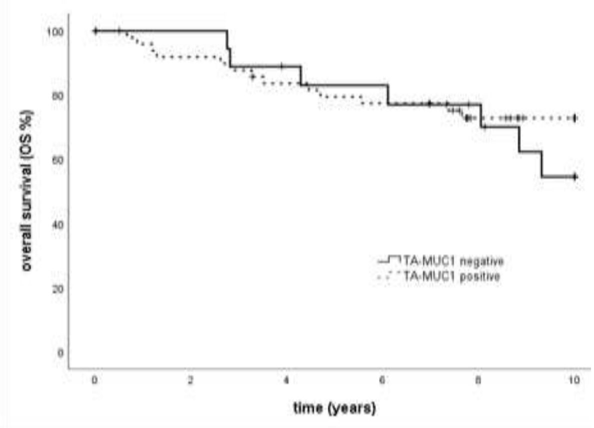

(**a**)

Siglec-8 high

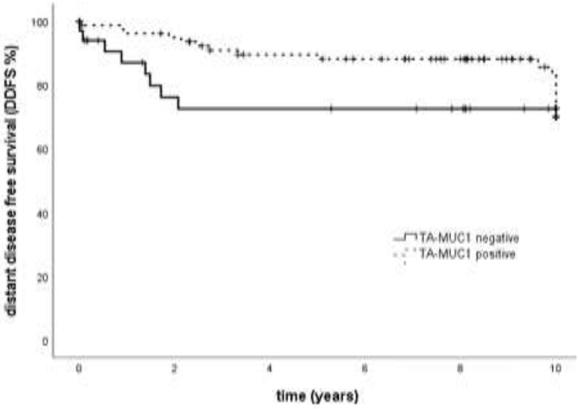

(**b**)

Siglec-8 low

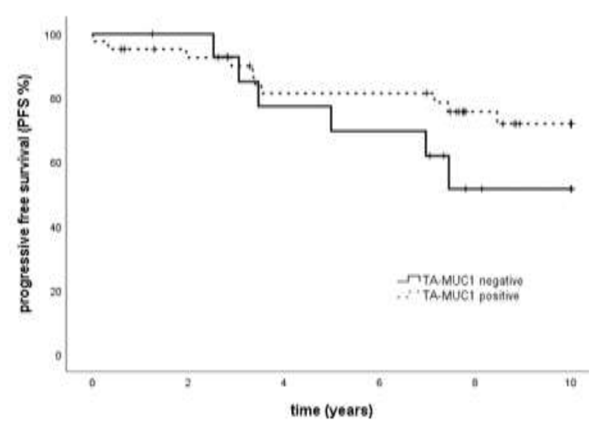

(**c**)

Siglec-8 high

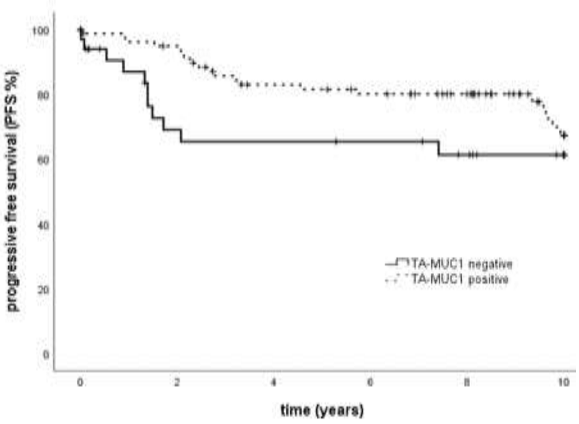

(**d**)

Figure SI 4. Not significant associations of TA-MUC1 expression to the clinical outcome in Siglec-8 low or Siglec-8 high subgroups.
Kaplan-Meier analysis of OS (a) ($p = 0.443$), DDFS (b) ($p = 0.307$) and PFS (c) ($p = 0.205$) and (d) ($p = 0.132$) in TA-MUC1-positive and negative-expressing tumors in Siglec-8 low-expressing (a, c), and Siglec-8 high expressing tumors (b, d).
